# Design of Nanostructured Surfaces for Efficient Condensation by Controlling Condensation Modes

**DOI:** 10.3390/mi14010050

**Published:** 2022-12-25

**Authors:** Qi Che, Fenghui Wang, Xiang Zhao

**Affiliations:** Bio-Inspired and Advanced Energy Research Center, School of Mechanics, Civil Engineering and Architecture, Northwestern Polytechnical University, Xi’an 710129, China

**Keywords:** vapor condensation, nanopillar surface, condensation mode, condensation rate, molecular dynamics simulation

## Abstract

To meet the different needs of various industrial fields, it is of great application value to find a feasible method for controlling the condensation mode on the surface. Inspired by biological surfaces, tuning the surface structure and wettability is considered as a potential way to control the surface condensation behavior. Herein, the coupling effect of the geometric parameters and wettability distribution of the surface on the condensation process has been investigated systematically at the nanoscale. The results illustrate that the condensation mode is primarily determined by the nanopillar wettability when the nanopillars are densely distributed, while the substrate wettability dominates the condensation mode when the nanopillars are sparsely distributed. Besides, the effective contact area fraction is proposed, which more accurately reflects the influence of geometric parameters on the condensation rate of the nanopillar surface at the nanoscale. The condensation rate of the nanopillar surface increases with the increase of the effective contact area fraction. Furthermore, three surface design methods are summarized, which can control the condensation mode of water vapor on the surface into the dropwise condensation mode that generates Cassie-Baxter droplets, and this condensation process is very attractive for many practical applications.

## 1. Introduction

Vapor condensation on solid surfaces is a common natural phenomenon, especially in mass and heat transfer processes [1]. In recent years, due to the increasing problems of regional water scarcity [2] and high energy consumption of heat exchange equipment [3], effective control of the condensation process has become an urgent demand in the fields of water harvesting [4], heat exchange [5], desalination [6], and anti-icing [7]. The vapor condensation process on solid surfaces often exhibits two condensation modes, filmwise condensation [8] and dropwise condensation [9]. It has been shown that surface wettability has a significant effect on the condensation mode for flat surfaces. Generally, the hydrophilic surface is conducive to the nucleation and growth of condensate. Due to the strong interaction between the surface and the water molecules, the condensate tends to become a water film covering the surface and is difficult to be removed. That is, the condensation mode of water vapor on the hydrophilic surface is often manifested as filmwise condensation. In contrast, the condensation mode on hydrophobic surfaces is usually dropwise condensation. Specifically, condensates are slower to nucleate and grow, but the droplets formed by vapor condensation are easily removed from the surface. The shedding of condensed droplets refreshes the surface, which makes the condensation process cyclic. Hence, the dropwise condensation could potentially offer orders of magnitude higher condensation efficiency than that of the filmwise condensation [10]. Meanwhile, the dropwise condensation exhibits five–seven times higher heat-transfer coefficient than that of filmwise condensation [9]. Additionally, for rough surfaces, the dropwise condensation is further divided into two types, corresponding to two wetting states of the condensed droplets (Wenzel state or Cassie-Baxter state). It is noteworthy that condensed droplets in different wetting states possess different abilities to detach from the surface. Since the droplet in the Cassie-Baxter state is easily dislodged from the surface, it is the ideal condensate in most applications. As a result, the condensation process corresponding to the dropwise condensation mode that generates Cassie-Baxter droplets is very attractive for many practical applications [4,5,7]. Since the form of condensation product has a significant impact on the overall performance of condensation, it is of great application value to find a feasible control method of the condensation mode on the surface to meet the needs of the different industrial fields mentioned above [11].

In the biosphere, many biological surfaces exhibit exceptional wettability due to the unique design of micro-nano structures and the intrinsic chemistry of the material, resulting in various advanced functions [12,13,14]. Among the numerous biomimetic research, studies of vapor condensation behavior on biological surfaces have inspired the design of artificial functional surfaces. For example, Zheng et al., in situ, studied the dynamic suspending behavior of microdroplets on lotus leaves at the micro- and nano-levels by observing the water condensation process through environmental scanning electron microscopy [15]. During water condensation, a gradient of wettable micro- and nano-structure is formed along the exterior surface of micropapillae (including nanohairs), which keeps the condensed microdroplets on the lotus leaves in the Cassie-Baxter state. In contrast, although the petals of red roses also have hierarchical micro- and nano-structures (densely arranged micropapillae on the surface and many nanofolds on each papillae top), water droplets are not in the Cassie-Baxter state on the petals [16]. The surface structures of the petals provide a high adhesive force with water, unlike the lotus leaf. Besides, the compound eyes of the mosquito *C. pipiens* exhibited striking superhydrophobic antifogging properties, resulted from the smart design of elaborate micro- and nano-structures (hexagonally non-close-packed nipples with average diameters of 101 nm and hexagonally close-packed ommatidia with a diameter of ca. 26 μm) [17]. Thus, mosquitoes maintain clear vision in a humid environment. The biomimetic studies mentioned above show that, although many biological surfaces are superhydrophobic, the difference in their surface microstructures gives rise to different condensation processes. That is, the structure parameters of the surface significantly affect the condensation mode on the surface. On the other hand, for some beetles in the Namib Desert, their efficient condensation property is endowed by the bumpy surface of their backs, which consists of alternating wax-coated (hydrophobic) and non-waxy (hydrophilic) regions [18]. This special surface wettability allows the beetles to collect drinking water from the fog-laden wind. Condensed droplets form on the hydrophilic top and roll down the hydrophobic valley to the insect’s mouthparts. Likewise, a clever design of dual wettability is also found on the young leaves of the *Phyllostachys aurea* bamboo, which is conducive to vapor condensation and directional transport [19]. This unique surface wettability can be attributed to a combination of the chemical and physical differences between the leaf margin and middle. The two biomimetic studies mentioned above demonstrate that the surface wettability distribution also has a strong influence on the condensation process. In addition, for Namib Desert beetles, Park et al. presented an unconventional interpretation of the role of the beetle’s bumpy surface geometry in promoting condensation [20]. Their theoretical model and experimental results demonstrated that the specific geometry of convex millimeter-sized surface structures alone could facilitate condensation. The review of these biomimetic studies suggests that tuning the structure and wettability distribution of the surface is a potential way to control condensation behavior on surfaces.

Recently, a variety of biomimetic surfaces are designed and the vapor condensation behavior on these surfaces is studied. Based on experimental observations, the effects of surface texture [21,22,23,24] and wettability distribution [25,26,27] on the condensation process are revealed. It is worth noting that, through imaging experiments, Enright et al. studied the condensation process on surfaces with different structure length scales (100 nm to 10 μm) and different wetting properties [24]. They found that the local energy barrier is the key to understanding the growth process and identified the role of nucleation density on the morphology of condensed droplet. Additionally, a regime map was developed to illustrate the effects of surface-structure length scale and energy on the emergent droplet morphology. It was deduced that the precise control of surface structure and chemistry could manipulate the condensation process. However, the current experimental studies are mostly macroscopic observations of condensed droplets with diameters ranging from several microns to several millimeters. The real-time characterization ability for microscopic droplet nucleation and growth is still lacking [24,27], despite significant advances in microscopic optics and environmental scanning electron microscopy imaging [28]. Therefore, the existing experimental methods are of limited help in understanding the effect of surface parameters on the initial stage of condensation. With the rapid development of computational capabilities, molecular dynamics (MD) simulation is used to understand the microscopic mechanism of the condensation nucleation on the surface by simulating the vapor condensation process at the nanoscale (usually refers to structures with a length scale of 1–100 nm) [29,30,31,32]. Sheng et al. [33] studied the nucleation processes of vapor condensation in a relatively large timescale to illustrate how the initial droplets develop and revealed the formation mechanisms of different condensation modes and the transition mechanism between them. Our previous work [34] investigated the condensation process on the substrate with various flexibilities, and the result showed that substrate flexibility has a significant effect on both the condensation mode and the heat transfer performance. Hiratsuka et al. [35] analyzed the impact of the wall nanostructure and adsorption force on the contact angle of droplets and condensation, and they found the contact angle and condensation behavior depended on the microfabrication shape and size of the wall. Besides, Gao et al. [36] investigated the condensation process on nanopillar surfaces with different wettability and found that the hybrid nanopillar surfaces exhibited better heat and mass transfer performance compared with other homogeneous nanopillar surfaces. That is, the condensation process can be controlled by a pre-designed surface wettability distribution. Ding et al. [37] studied the dewetting transition of water on surfaces during vapor condensation and verified that nanocone surfaces with an appropriate hybrid wettability ratio providing better dewetting performance without scarifying condensation performance.

However, while much effort has been devoted to studying condensation processes on various structured surfaces using MD simulation, there is still a lack of sufficient understanding of droplet nucleation and wetting behavior on nanostructured surfaces with hybrid wettability. In addition, the coupling effect of surface structure parameters and surface wettability distribution on the condensation process also needs to be investigated in detail at the nanoscale. In this work, the condensation processes of water vapor on various surfaces are probed using MD simulations. The effects of the geometric parameter and wettability distribution of the nanostructured surfaces on the condensation process are systematically investigated. The geometric parameter studied here refer to the spacing between nanostructures. Since the effect of individual nanostructure morphology on condensation is not investigated in this study, simple nanopillar surfaces are chosen as typical nanostructured surfaces for vapor condensation study. The results show that the condensation mode and condensation rate of water vapor on the nanopillar surface are significantly influenced by the surface geometric parameters and wettability. Besides, a parameter named effective contact area fraction is proposed in this work. In comparison with the surface solid fraction commonly used in previous analyses, the effective contact area fraction can more accurately reflect the influence of geometric parameters on the condensation rate of the nanopillar surface at the nanoscale. Furthermore, for efficient condensation, three surface design methods are summarized, which can control the condensation mode of water vapor on the surface into the dropwise condensation mode that generates Cassie-Baxter droplets. This work clarifies the relationship between condensation mode and surface parameters when vapor condenses on nanostructured surfaces and proves that tuning structure and wettability distribution of the surface is an effective way to control condensation behavior on the surface.

## 2. Models and Methods

**Figure 1:**
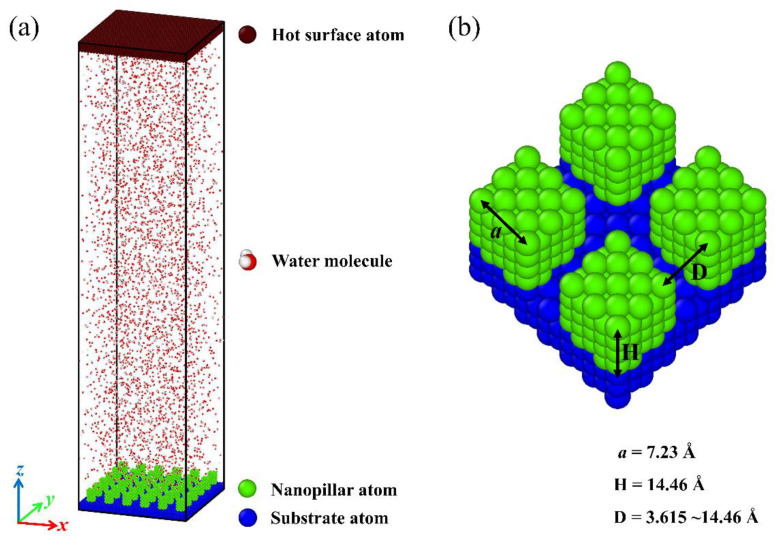
(**a**) An overview of the simulation domain. (**b**) Schematic diagram for parameters of nanopillar surface.

The simulation model is composed of two surfaces and four thousand water molecules in a computational region of size 130 Å × 130 Å × 564 Å. Periodic boundary conditions are applied in all three spatial dimensions. The initial configuration of the system is shown in Figure 1a. As the condensation material, water molecules are initially distributed in a discrete manner. Specifically, a cold nanopillar surface is placed at the bottom of the system to serve as a substrate for water vapor condensation, and a hot flat surface is placed at the top of the system to ensure that all water molecules can condense on the topside of the nanopillar surface. Both the flat surface and the nanopillar surface are composed of Cu-like atoms with a face-centered cubic (FCC) crystal structure, and the lattice constant equals 3.615 Å. The nanopillar surface is composed of a smooth substrate and the nanopillars grown on the substrate. The structural parameters are defined as shown in Figure 1b, and the detailed description about these geometric parameters are given in the Supplementary Materials. 

A pairwise Lennard-Jones (LJ) potential is used to simulate the interatomic interaction between Cu-like atoms. The standard 12-6 LJ potential is given as

(1) $$U_{\mathrm{LJ}}\;=\;4\varepsilon_{\mathrm{ij}}[{\left(\sigma_{\mathrm{ij}}/r_{\mathrm{ij}}\right)}^{12}\;-\;{\left(\sigma_{\mathrm{ij}}/r_{\mathrm{ij}}\right)}^{6}],r\;<\;r_{c}$$

where *r_ij_* is the distance between a pair of atoms, *ɛ_ij_* is the characteristic energy parameter, and *σ_ij_* is the distance parameter. In this work, the cutoff distance *r_c_* is set as 10 Å. Additionally, a harmonic potential is applied to all Cu-like atoms to maintain the geometrical shape of two surfaces during the simulation [29,38], given as

(2) $$E_{H}(r)\;=\;K{\left(r\;-\;r_{0}\right)}^{2}$$

where *K* is the spring constant and *r*_0_ is the equilibrium position of the atom. The TIP4P-Ew water model is used for water molecules in the simulations [39]. The bond and the angle of the water molecular are constrained by using the SHAKE algorithm [40]. The force field parameters of surface atoms and water molecules used in this work are given in the Supplementary Materials.

The LJ potential can be used to describe the interaction between the nanopillar surface and water molecule. The setting of the energy parameter (*ε*_water-Cu_) determines the surface wettability. Here, two values of *ε*_water-Cu_ are mainly used to represent the different wettability surfaces, 0.25 and 0.4 kcal mol^−1^, respectively. The contact angle (*θ*) of a water droplet on a flat surface with the same parameters *ε*_water-Cu_ is obtained by the method used in the previous work [34]. The results show that the contact angle is 98° when *ε*_water-Cu_ is 0.25 kcal mol^−1^, while it becomes 38° when *ε*_water-Cu_ is 0.4 kcal mol^−1^. The details of the method and the morphology of droplets are given in the Supplementary Materials. Clearly, the nanopillar surface will exhibit different wettability distributions when the energy parameters between the nanopillar atoms and water molecules or between the substrate atoms and water molecules are changed. 

All MD simulations are performed using the LAMMPS package [41]. The standard velocity Verlet algorithm is used for the numerical integration of Newton’s equation of motion, and the time step is set to 1 fs. Furthermore, the cutoff distance for the computation of short-range coulombic interactions is set as 12 Å, and the long-range coulombic interactions are computed using the particle–particle particle–mesh (PPPM) method with a precision of 10^4^ [42]. In order to simulate the condensation process of high temperature water vapor on the nanopillar surface, each simulation requires a two-stage process. In the first stage, to ensure a rational simulation structure, the conjugate gradient algorithm is used for the energy minimization of the initial system; then, the system is carried out in the canonical ensemble (NVT) at 500 K for 0.2 ns to reach an equilibrium state. The configuration of the simulation system obtained at this stage will be used as the initial configuration for the subsequent condensation process. In the second stage, the nanopillar surface is controlled at 300 K by the Langevin thermostat and the temperature control of the water molecules is released, while the flat surface remains at 500 K. The processing time of this stage is 5 ns to simulate the condensation process of high temperature water vapor on the nanopillar surface.

This work aims at testing the condensation of water vapor on the nanostructured surfaces and assesses the effect of differences in surface chemistry and structure on the condensation process. For this purpose, a series of nanopillar surfaces is constructed by changing the geometric parameters of nanostructures on the surface and the surface wettability distribution. To exclude the influence of the single nanopillar size on the condensation process, the dimensions of the square nanopillar in each model are fixed, with width *a* = 7.23 Å and height H = 14.46 Å, as shown in Figure 1b. Here, the geometric parameter of nanostructures on the surface used for investigation is the interpillar spacing D. Obviously, the surface morphology varies with the change of the interpillar spacing. Meanwhile, the nanopillar surfaces with different wettability distributions are defined by varying the energy parameters between the nanopillar atoms and water molecules or between the substrate atoms and water molecules, as described above. The subscript notation for nanopillar wettability, substrate wettability, and interpillar spacing is described in Table 1. Accordingly, each nanopillar surface studied in this work can be denoted as P_i_S_j_D_k_. For example, P_0.25_S_0.4_D_1_ represents the nanopillar surface with hydrophobic nanopillars and hydrophilic substrate, and the interpillar spacing is 3.615 Å.

## 3. Results and Discussions

### 3.1. Condensation on Nanopillar Surfaces

The condensation processes of water vapor on nanopillar surfaces with different geometric parameters and wettability distributions are investigated by MD simulation. The results show that the vapor condensation is significantly affected by the surface structure and wettability. Meanwhile, three typical condensation modes are observed on nanopillar surfaces. In this section, the representative condensation processes corresponding to various condensation modes are presented first. Then, the final morphology of the condensate on each nanopillar surface and the potential energy between water molecules and various nanopillar surfaces are shown, and the effects of interpillar spacing and wettability distribution on the condensation mode are discussed.

#### 3.1.1. Condensation Process of Three Typical Condensation Modes

**Figure 2:**
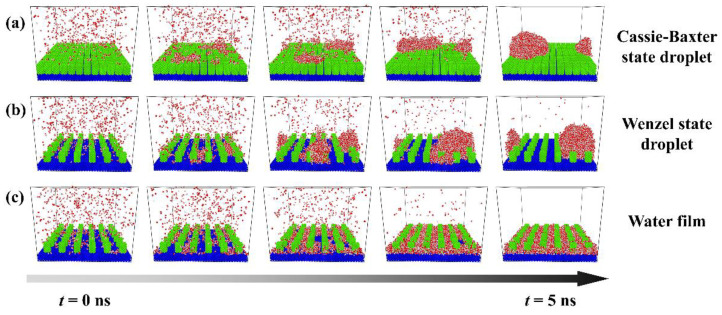
Condensation processes of three typical condensation modes on the nanopillar surface. (**a**) Cassie-Baxter DWC mode observed on the surface P_0.4_S_0.4_D_1_; (**b**) Wenzel DWC mode observed on the surface P_0.25_S_0.25_D_3_; (**c**) FWC mode observed on the surface P_0.4_S_0.4_D_3_.

The evolutions of each typical condensation modes are shown in Figure 2, and these figures only describe the space close to the nanopillar surface. Figure 2a shows the condensation process on the surface of P_0.4_S_0.4_D_1_. At first, water molecules condense into small clusters on the surface randomly. Then, some clusters grow and coalesce with adjacent clusters to evolve into a nanoscale droplet. This nanodroplet continues to grow by absorbing water molecules and ends up in the Cassie-Baxter state on the surface. So, this condensation process can be defined as the dropwise condensation mode that generates Cassie-Baxter droplets, denoted as Cassie-Baxter DWC mode. Figure 2b shows the condensation process on the surface of P_0.25_S_0.25_D_3_. It is similar to the case in Figure 2a, except that the water molecules eventually condense into a Wenzel state droplet on the surface. This condensation process can be defined as the dropwise condensation mode that generates Wenzel droplets, denoted as Wenzel DWC mode. Figure 2c shows the condensation process on the surface of P_0.4_S_0.4_D_3_. Initially, water molecules form dense clusters on the surface. As the clusters merge and grow, the condensed water molecules gradually fill in the gaps between the nanopillars and eventually evolve into a water film overlying the surface. This condensation process can be defined as the filmwise condensation (FWC) mode.

**Figure 3:**
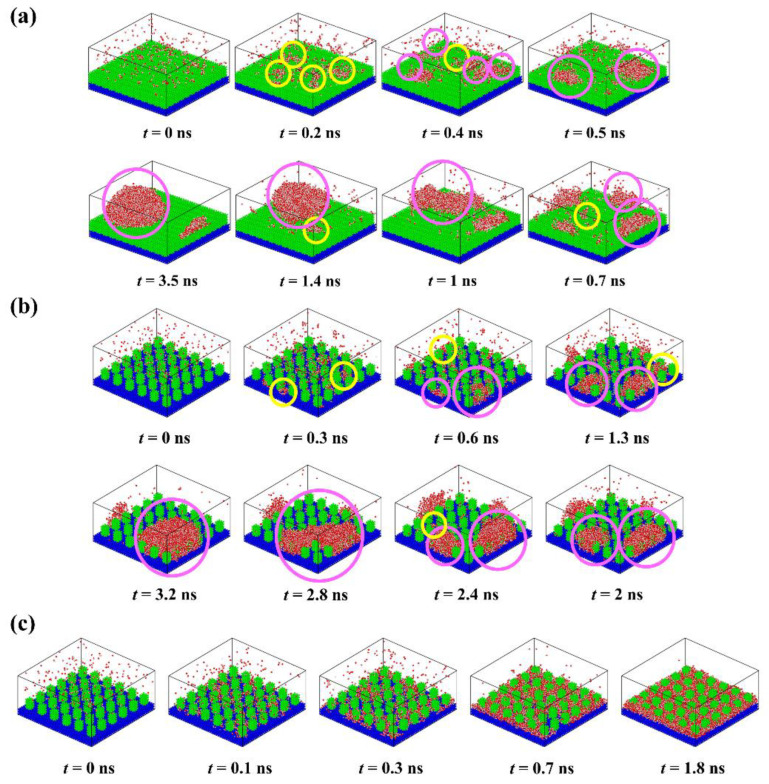
Time-lapse images of the condensation process on different nanopillar surface. (**a**) P_0.4_S_0.4_D_1_; (**b**) P_0.2_5S_0.25_D_3_; (**c**) P_0.4_S_0.4_D_3_. (“
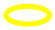
” represents newly formed water clusters; “
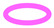
” represents pre-existing water clusters).

To understand the condensation behavior of water vapor on rough surfaces, detailed descriptions of the formation process of each typical condensate are provided, as shown in Figure 3. At the beginning of the simulation, the molecules in the water vapor collide with each other due to random thermal motion. Some water molecules close to the surface collide with surface atoms and thus form clusters deposited on the surface. The water molecules in these clusters lose some kinetic energy, the corresponding thermal energy was absorbed by the surface. Subsequently, the clusters will become larger as more water molecules aggregate, or they may become smaller as the water molecules within them return into the water vapor, which exhibits the instability of small clusters. For the surface P_0.4_S_0.4_D_1_ (Figure 3a), water clusters are deposited on top of the nanopillars to form randomly distributed nuclei because water molecules cannot enter the narrow gaps when the interpillar spacing is D_1_. With the growth and irregular thermal motion of the nuclei, they merge with adjacent nuclei and grow up into nanodroplets. Afterwards, the condensed droplets grow up into Cassie-Baxter droplets by absorbing more water molecules or merging with newly condensed nuclei on the surface. Due to the limited number of water molecules in the simulated domain, condensation process ends when all water molecules have condensed on the surface. For the surface P_0.25_S_0.25_D_3_ (Figure 3b), water molecules are preferentially deposited in the gaps between the nanopillars, which is consistent with the conclusions obtained by Xu et al. and Du et al. using the classical nucleation theory [43,44]. The nuclei grow up by the addition of water molecules, but their movement is restricted by the nanopillars. As the nuclei grow, they merge with adjacent nuclei and grow up into nanodroplets. After growth and repeated coalescence, the nanodroplets grow up into Wenzel droplets. For the surface P_0.4_S_0.4_D_3_ (Figure 3c), water molecules are rapidly deposited in the gaps and form dense nuclei due to the strong attraction of the hydrophilic surface to water molecules. The nuclei easily merge with each other and form a water film filled in the gaps with a thickness of about two–three water molecules. Then, the water film thickens by absorbing water molecules. These findings show that the condensation process involves cluster formation, nucleation, and the emergence of nanodroplets (or water films). Additionally, snapshots of condensation process on all the proposed nanopillar surfaces are given in the Supplementary Materials (Figures S3–S7).

#### 3.1.2. Condensation on the Nanopillar Surfaces with Uniform Wettability

**Figure 4:**
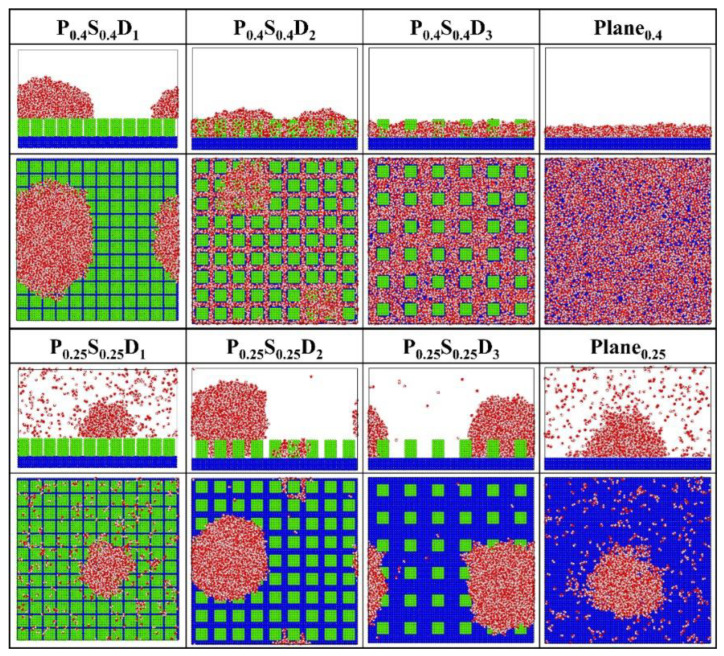
The snapshot of the condensate formed on the surfaces with uniform wettability at the end of the simulation.

Figure 4 presents the final morphology of the condensate on the nanopillar surfaces with uniform wettability, and on the flat surface with corresponding wettability (denoted as Plane_i_) for comparison. For flat hydrophilic surfaces (Plane_0.4_), the condensed water molecules grow into a water film that completely covers the surface. Similarly, the FWC mode is also observed on the hydrophilic nanopillar surfaces (P_0.4_S_0.4_) with interpillar spacing D_2_ and D_3_. However, when the interpillar spacing is reduced, that is, in the case of P_0.4_S_0.4_D_1_, the water molecules condense into Cassie-Baxter state droplets on the surface. This is an interesting result because dropwise condensation occurs on a completely hydrophilic surface. However, for flat hydrophobic surfaces (Plane_0.25_), the DWC mode is observed but not all water molecules in the space condensed on the surface. For the hydrophobic nanopillar surfaces (P_0.25_S_0.25_), almost all water molecules condensed into Wenzel state droplets when the interpillar spacing is D_2_ and D_3_. However, in the case of P_0.25_S_0.25_D_1_, only a few water molecules condensed on the surface, and the condensed droplet is in the Cassie-Baxter state. Accordingly, compared to the condensation process on Plane_0.25_, adding sparse hydrophobic nanopillars on the hydrophobic surface accelerates the condensation of water molecules, while the addition of dense hydrophobic nanopillars delays the condensation of water vapor on the hydrophobic surface to some extent. That is, the addition of dense hydrophobic nanopillars enhances the anti-condensation ability of the hydrophobic surface. Overall, these findings suggest that the surface structure significantly influences the condensation mode of water vapor on the nanostructured surface.

**Figure 5:**
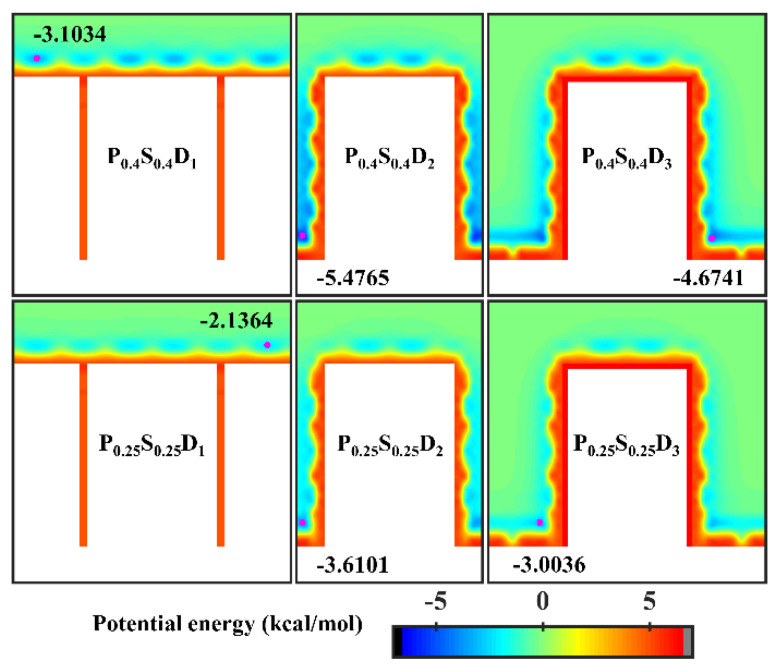
Equipotential curves of potential energy between water molecule and the nanopillar surfaces with uniform wettability. The minimum values of the potential energy are given in the figure and their positions are marked with a pink dot.

To further study the effects of the geometric parameters and wettability distribution of the nanopillar surface on the condensation mode, the preferential condensation location of water molecules during condensation and the influence of the surface on the condensate morphology are analyzed through potential energy. According to the standard 12-6 LJ potential, the potential energy between a single water molecule and various nanopillar surfaces is calculated. Figure 5 presents the equipotential curves of potential energy in the cases of nanopillar surfaces with uniform wettability. As one can see, the surface geometric parameters greatly affect the potential energy distribution on the nanopillar surface and thus the condensation process of water vapor on the nanostructured surface. When the interpillar spacing is D_1_, the extremely high energy barrier prevents water molecules from entering the gaps between the nanopillars, which causes the condensed droplets to be in the Cassie-Baxter state. Since the potential energy near the hydrophilic nanopillar surface is lower than that near the hydrophobic surface, more water molecules condense on the surface P_0.4_S_0.4_D_1_. However, as the interpillar spacing increases (D_2_ and D_3_), the energy barrier in the gaps disappears and the gaps become negative potential energy, so the water molecules are inclined to condense in the gaps. As shown in Figure 2b,c, water molecules randomly condense into multiple small clusters in the gaps at the onset of condensation, and then the clusters merge and grow. Due to the low potential energy near the hydrophilic nanopillar surface, the condensed water molecules eventually evolve into a water film overlying the surface. However, the hydrophobic nanopillar surface does not attract water molecules as strongly as the hydrophilic surface, so the water clusters gradually form Wenzel droplets immersed in the gap as they merge and grow. It should be noted that the dimensions of the white region (referring to the nanopillar and the substrate) in all energy curves in this work have considered the atomic radius of Cu, ~ 1.28 Å. Based on this, the nanopillar width is ca. 9.79 Å in the figure.

**Figure 6:**
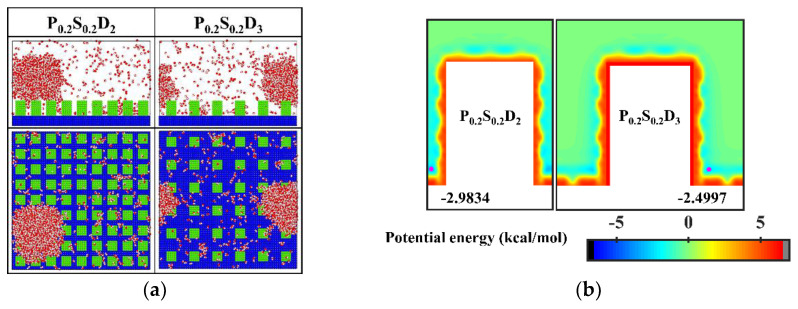
(**a**) The snapshot of the condensate formed on the surfaces at the end of the simulation in the cases of P_0.2_S_0.2_D_2_ and P_0.2_S_0.2_D_3_. (**b**) Equipotential curves of potential energy between water molecule and the nanopillar surfaces (P_0.2_S_0.2_D_2_ and P_0.2_S_0.2_D_3_). The minimum values of the potential energy are given in the figure and their positions are marked with a pink dot.

It is well known that the condensation process corresponding to the Cassie-Baxter DWC mode has a theoretically high condensation efficiency. For the nanopillar surface with uniform wettability, the Cassie-Baxter DWC mode is only observed when the interpillar spacing is D_1_. As mentioned above, it is difficult for water molecules to enter the gaps between the nanopillars due to the small spacing when the interpillar spacing is D_1_. Thus, the condensed droplet is in the Cassie-Baxter state. When the interpillar spacing increases to D_2_ or D_3_, no Cassie-Baxter state droplets can be observed on surfaces throughout the condensation process. In general, the wetting state of the droplet on the roughness surface depends on its surface tension as well as the interaction with the rough surface. It has been demonstrated that nanodroplets on rough surfaces can be in the Wenzel state or the Cassie-Baxter state depending on the geometric parameters of the nanostructures and the interaction parameters between water molecules and surface atoms [35,45]. Here, to condense water vapor into Cassie-Baxter droplets on the nanopillar surfaces with large interpillar spacing (relative to D_1_), a new interaction parameter, 0.2 kcal mol^−1^, is added to represent a stronger hydrophobicity (the contact angle is 110° when *ε*_water-Cu_ is 0.2 kcal mol^−1^, and the morphology of droplets on the corresponding flat surface is given in the Supplementary Materials). Using this interaction parameter, two nanopillar surfaces with stronger hydrophobicity are modeled to complement this study, denoted as P_0.2_S_0.2_D_2_ and P_0.2_S_0.2_D_3_, respectively. The final morphology of the condensate in the supplementary simulations and the equipotential curves of potential energy in these cases are shown in Figure 6. Obviously, the condensed droplets are in the Cassie-Baxter state on both surfaces. From the perspective of potential energy, when the interpillar spacing is D_2_ or D_3_, the potential energy at the bottom of the gap between the nanopillar on the surface with energy parameter of 0.2 kcal mol^−1^ is elevated by 17% compared to that of the surface with energy parameter of 0.25 kcal mol^−1^. The elevation of potential energy indicates the bottom of the gap becomes less attractive to water molecules. Therefore, when condensation occurs on the nanopillar surface with energy parameter of 0.2 kcal mol^−1^, the condensed nanodroplets will move away from the bottom of the gap due to thermal motion. According to the simulation results in this section, reducing the interpillar spacing or the surface energy are beneficial to adjusting the condensation mode of water vapor on the surface into the dropwise condensation mode that generates Cassie-Baxter droplets.

#### 3.1.3. Condensation on the Nanopillar Surfaces with Hybrid Wettability

**Figure 7:**
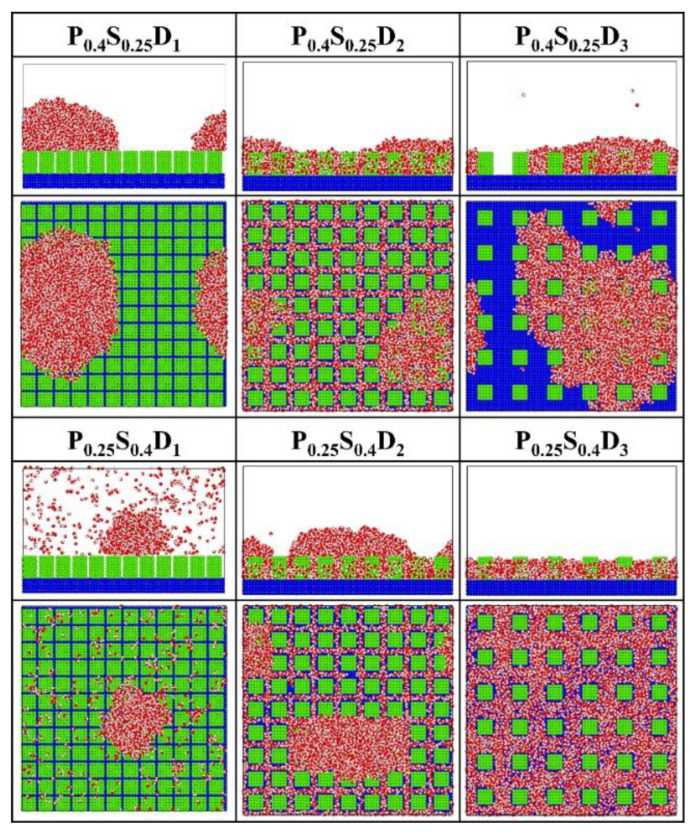
The snapshot of the condensate formed on the surfaces with hybrid wettability at the end of the simulation.

Figure 7 presents the final morphology of the condensate on the nanopillar surfaces with hybrid wettability. When the substrate is hydrophobic and the nanopillars are hydrophilic, the DWC mode is observed on the surfaces with interpillar spacing D_1_ and D_3_, where the condensed droplets are in the Cassie-Baxter state on the surface P_0.4_S_0.25_D_1_ and in the Wenzel state on the surface P_0.4_S_0.25_D_3_. However, the vapor condensation on the surface with interpillar spacing D_2_ shows as the FWC mode. In contrast, when the substrate is hydrophilic and the nanopillars are hydrophobic, a condensed droplet in the Cassie-Baxter state is observed on the surface with interpillar spacing D_1_, but the condensation is very slow (only a small amount of water molecules condensed on the surface at 5 ns); meanwhile, the vapor condensation on the surfaces with interpillar spacing D_2_ and D_3_ is in the FWC mode. Compared to the condensation results on the nanopillar surfaces with uniform wettability, it is demonstrated that, for the nanopillar surfaces with hybrid wettability, when the nanopillars are densely distributed (in the case of D_1_), the condensation mode is primarily determined by the wettability of the nanopillars; when the nanopillars are sparsely distributed (in the case of D_3_), the substrate wettability mainly affects the condensation mode; and when the interpillar spacing is D_2_, the priority is unclear.

**Figure 8:**
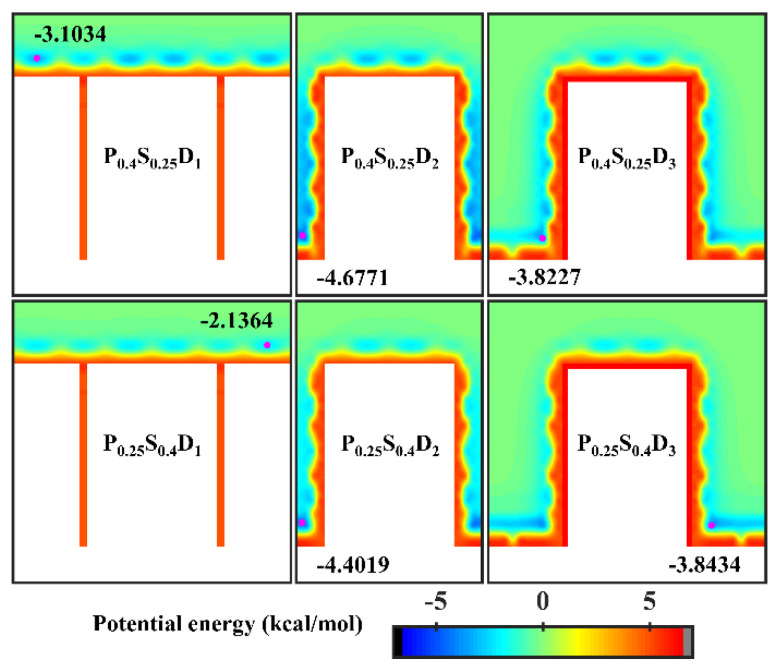
Equipotential curves of potential energy between water molecule and the nanopillar surfaces with hybrid wettability. The minimum values of the potential energy are given in the figure and their positions are marked with a pink dot.

In order to analyze the above results, the equipotential curves of potential energy in the cases of nanopillar surfaces with hybrid wettability are calculated, as shown in Figure 8. When the interpillar spacing is D_1_, the potential energy in the contact area between the surface and the water molecules is mainly determined by the wettability of the nanopillars. When the interpillar spacing is D_3_, because the contact area between the substrate and the water molecules becomes larger, the potential energy at the bottom of the gap profoundly affects the ability of the gap to confine the condensate, that is, the condensation mode in this case is determined by the substrate wettability. When the interpillar spacing is D_2_, both the wettability of the nanopillars and the substrate wettability have a significant effect on the potential energy distribution in the narrower gaps, so they do not have a clear priority of influence on the condensation mode.

### 3.2. Effect of Nanopillar Surface Parameters on Condensation Rate

To quantitatively compare the condensation rate on different nanopillar surfaces, the number of water molecules in the largest condensed cluster (*N*_max_) on the surface is counted. Water molecules are identified as in the same cluster when they are within 1.5 *σ*_O–O_ [46], where *σ*_O–O_ = 3.16435 Å, the distance parameter defined in the TIP4P-Ew model.

#### 3.2.1. Interpillar Spacing

**Figure 9:**
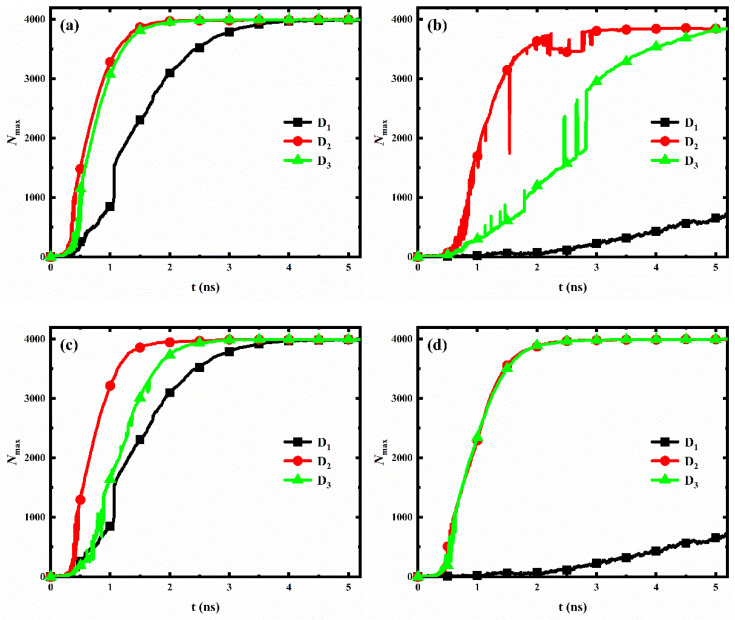
The evolution of the number of water molecules in the largest size cluster for surfaces with different interpillar spacing. (**a**) Hydrophilic nanopillar surfaces; (**b**) hydrophobic nanopillar surfaces; (**c**) nanopillar surfaces with hydrophobic substrate and hydrophilic nanopillars; (**d**) nanopillar surfaces with hydrophilic substrate and hydrophobic nanopillars.

For each wettability distribution, the evolution of the number of water molecules in the largest size condensed clusters on the nanopillar surfaces with different interpillar spacing is shown in Figure 9. Clearly, for nanopillar surfaces with uniform wettability (Figure 9a,b) or nanopillar surfaces with hydrophobic substrate and hydrophilic nanopillars (Figure 9c), the condensation rate of the nanopillar surface has the same trend with the changed interpillar spacing, i.e., *v*_2_ > *v*_3_ > *v*_1_, where *v*_i_ denotes the condensation rate of nanopillar surfaces with interpillar spacing D_i_. However, for nanopillar surfaces with hydrophilic substrate and hydrophobic nanopillars (Figure 9d), the condensation rates are similar when the interpillar spacing is D_2_ and D_3_, and significantly slower when the interpillar spacing is D_1_, i.e., *v*_2_ ≈ *v*_3_ > *v*_1_.

However, the variation of the condensation rate observed in our simulation results differs from the general conclusions obtained by previous work. Gao et al. [36] analyzed the condensation on nanopillar surfaces with different solid fractions ϕ ($\phi \;=\;a^{2}/{\left(a\;+\;D\right)}^{2}$, where *a* is the width of nanopillar and D is the interpillar spacing, as shown in Figure 1b), and they found that the condensation rate of nanopillar surface decreases with the decrease of surface solid fraction. According to their conclusion, the condensation rate of our model should be consistent with *v*_1_ > *v*_2_ > *v*_3_, which is clearly inconsistent with our observations mentioned above. Here, to accurately reflect the influence of geometric parameters on the condensation rate of the nanopillar surface at the nanoscale, a parameter is proposed, namely the effective contact area fraction (η). This parameter refers to the ratio of the actual surface area where the nanopillars in contact with water molecules to the total geometric surface area of the nanopillar surface. Based on the geometric parameters of the nanopillar surface defined in Figure 1b, $\eta_{D_{i}}\;=\;\left(a^{2}\;+\;4aH\right)/[{\left(a\;+\;D_{i}\right)}^{2}\;+\;4aH]$. However, water molecules cannot enter the gaps between the nanopillars when the interpillar spacing is D_1_. So, $\eta_{D_{1}}\;=\;a^{2}/[{\left(a\;+\;D_{1}\right)}^{2}\;+\;4aH]$. The surface solid fraction, the effective contact area fraction and the surface roughness of nanopillar surfaces with different interpillar spacing are given in Table 2. Clearly, the effective contact area fraction for the nanopillar surfaces defined in the present work have the following relation: $\eta_{D_{1}}\;<\;\eta_{D_{3}}\;<\;\eta_{D_{2}}$, which is consistent with the observed variation law of the condensation rate. This suggests that, at the nanoscale, the effective contact area fraction can more accurately reflect the influence of geometric parameters on the condensation rate of the nanopillar surface compared to the surface solid fraction. Specifically, the condensation rate of the nanopillar surface increases with the increase of the effective contact area fraction. Notably, although the surface P_0.4_S_0.4_D_2_ has a larger effective contact area compared to that of the surface P_0.4_S_0.4_D_3_, it does not show a significantly faster condensation rate, as shown in Figure 9a. This might be due to the uncertainty in the analysis data. As was well known, the molecular dynamics method is a deterministic method. Once the initial configuration and velocity are determined, the trajectory of the molecules over time is also determined. However, during the actual simulation, the trajectories of the atoms in the same model may vary in multiple simulations due to differences in settings, algorithm selection, and computational equipment, while the structural characteristics and properties of the overall system remain basically stable. Since the analysis data in Figure 9 (*N*_max_) are sensitive to the atomic positions in the simulation, there is a degree of uncertainty in the statistics of this data. According to the effective contact area fraction, condensation on the surface P_0.4_S_0.4_D_2_ should be faster than that on the surface P_0.4_S_0.4_D_3_.

In general, the hydrophilic region possesses a faster condensation rate due to its strong interaction with water molecules. Therefore, for nanopillar surfaces with hydrophilic substrate and hydrophobic nanopillars, the substrate is considered to have a more significant effect on accelerating the condensation process than the nanopillars. Accordingly, although the surface P_0.25_S_0.4_D_2_ has a larger effective contact area fraction than the surface P_0.25_S_0.4_D_3_, the condensation rates of these two surfaces are close due to the fact that the surface P_0.25_S_0.4_D_3_ has a larger area of the exposed hydrophilic substrate. However, when the interpillar spacing is D_1,_ the dense hydrophobic nanopillars hinder the contact of water molecules with the hydrophilic substrate, which significantly reduces the condensation rate.

#### 3.2.2. Surface Wettability Distribution

**Figure 10:**
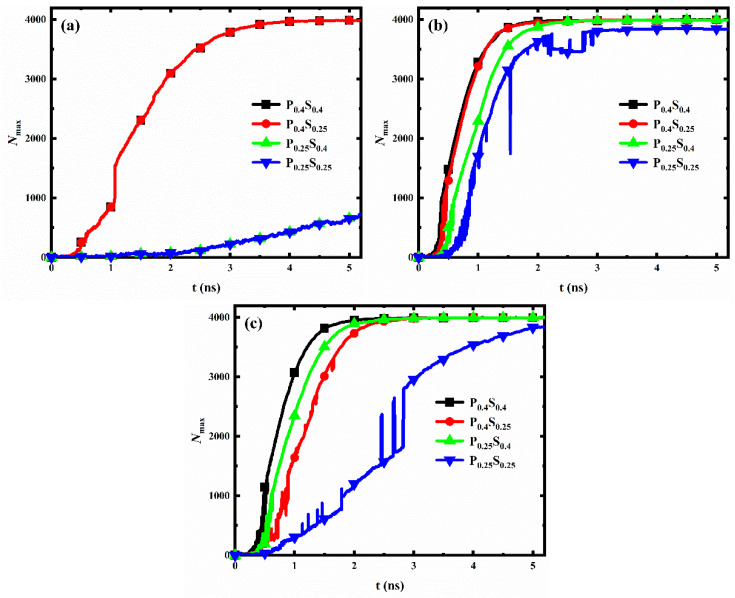
The evolution of the number of water molecules in the largest size cluster for nanopillar surfaces with different wettability distributions. The interpillar spacing of the surface is (**a**) D_1_; (**b**) D_2_; (**c**) D_3_.

For each interpillar spacing, the evolution of the number of water molecules in the largest size condensed clusters on the nanopillar surfaces with different wettability distributions is shown in Figure 10. When the nanopillar surface geometry is determined, condensation on the hydrophilic nanopillar surface is always the fastest at the initial stage of water vapor condensation, while condensation on the hydrophobic nanopillar surface is always the slowest. In addition, when the interpillar spacing is D_1_ or D_2_, the condensation rates of the nanopillar surfaces with hydrophobic substrate and hydrophilic nanopillars are greater than that of the nanopillar surfaces with hydrophilic substrate and hydrophobic nanopillars; however, the opposite is true when the interpillar spacing is D_3_.

As mentioned in Section 3.2.1, the hydrophilic region possesses a faster condensation rate than the hydrophobic region. When the interpillar spacing is D_1_ or D_2_, water vapor is mainly in contact with the nanopillars, so the nanopillar wettability mainly affects the condensation rate. Accordingly, the condensation rate of the surfaces with hydrophilic nanopillars is larger. However, when the interpillar spacing of the surface increased to D_3_, a larger area of the substrate is exposed to the space. The enlarged substrate area by reducing the nanopillar density allows the substrate to come into contact with more water molecules, which leads to a weaker effect of nanopillar wettability on condensation and an enhanced effect of substrate wettability on the condensation process. Thus, the condensation rate is greater on the surfaces with the hydrophilic substrate. Overall, the effect of wettability distribution on condensation rate differs for nanopillar surfaces with different geometric parameters.

### 3.3. Dewetting-Like Behavior during Condensation

**Figure 11:**
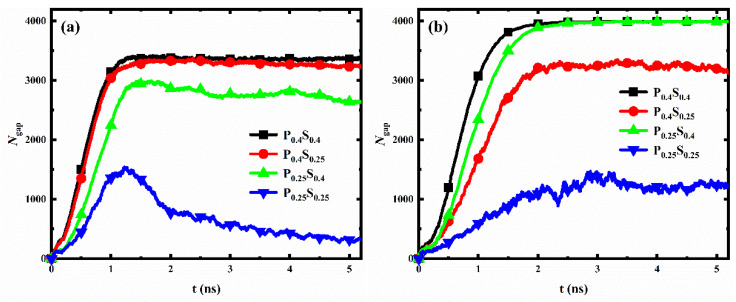
The evolution of the number of water molecules in the gaps between nanopillars for different nanopillar surfaces. The interpillar spacing of the surface is (**a**) D_2_; (**b**) D_3_.

The transition of droplets on a rough surface from the Wenzel state to the Cassie-Baxter state is called dewetting [47,48,49]. Usually, after the dewetting transition, the liquid in the gaps between surface structures will move to the top of the structures, resulting in a significant decrease in the number of liquid molecules in the gaps. Ding et al. [37] demonstrated that nanostructured surfaces (nanopillar with radius of 1 nm) with suitable geometric design and wettability modifications could exhibit better dewetting transition ability and condensation performance. Herein, the number of water molecules in the gaps between nanopillars (*N*_gap_) for different nanopillar surfaces is calculated and recorded, as shown in Figure 11 (since the water molecules cannot enter the structure gap when the interpillar spacing is D_1_, only the data for the surfaces with interpillar spacing D_2_ and D_3_ are displayed in the figure). With the progress of condensation, the *N*_gap_ for most of the nanopillar surfaces increases first and then remains almost constant. However, for the two nanopillar surfaces, P_0.25_S_0.25_D_2_ and P_0.25_S_0.4_D_2_, the *N*_gap_ first increases and then decreases as the simulation proceeds, which is similar to the trend when the dewetting transition occurs.

As mentioned in Section 3.1, the transition of the condensed droplet from the Wenzel state to the Cassie-Baxter state is not observed in our simulations. That is, the nanopillar surfaces modeled in this work all lack dewetting transition ability. However, for both surfaces, P_0.25_S_0.25_D_2_ and P_0.25_S_0.4_D_2_, a large number of water molecules enter the gaps in the early stage of condensation, and part of the condensed water molecules in the gaps moves to the top of the nanopillar as the condensation proceeds. This form of molecular motion is similar to what occurs during the dewetting transition, but there are still many water molecules in the gaps. Hence, this phenomenon in our simulation can be formulated as dewetting-like behavior. According to the results, two kinds of dewetting-like behavior are discovered. First, for the surface P_0.25_S_0.25_D_2_, a hydrophobic nanopillar surface, although the condensed droplet does not transition from the Wenzel state to the Cassie-Baxter state, the water molecules in the gaps decrease significantly as the droplet grows gradually. This is different from the case of other dropwise condensation processes that generate Wenzel droplets. Such as, no significant reduction of the *N*_gap_ is observed during the condensation for the surfaces P_0.25_S_0.25_D_3_ and P_0.4_S_0.25_D_3_. This dewetting-like behavior based on Wenzel droplets could effectively reduce condensate in the gaps between surface structures. On the other hand, for the surface P_0.25_S_0.4_D_2_, a surface with hydrophilic substrate and hydrophobic nanopillars, although the water molecules condense into a liquid film filling the gaps on surface, the water molecules in the gaps decrease slightly during subsequent simulation. This reduction of water molecules is not found in the other filmwise condensation processes in this work. This dewetting-like behavior based on liquid film reduces the condensate in the gaps to some extent. It should be noted that for the nanopillar surfaces where dewetting-like behavior is observed, when the interpillar spacing of the surface is changed while retaining the surface wettability distribution, no dewetting-like behavior occurred on the surface. This suggests that both the wettability distribution and the geometric parameters of the surface are decisive factors for the occurrence of the dewetting-like behavior. 

Wang et al. studied the spontaneous dewetting transition of nanoscale droplets on the nanopillar surface (nanopillar with diameters ranging from 0.68 and 1.76 nm) by molecular dynamics simulations [49]. They demonstrated that the droplet volume is the key to spontaneous dewetting transition of nanoscale droplets. There exists the critical volume of droplet, and the increase in droplet volume is beneficial to the dewetting transition on the same surface. For the surface P_0.25_S_0.25_D_2_, nanodroplets appear and grow up into Wenzel droplet. As condensation proceeds, the increase in droplet volume helps the droplet to change from the Wenzel state to the Cassie-Baxter state. However, the volume of the condensed droplet might not reach the critical value even at the end of condensation, no dewetting transition occurred. Since a large number of water molecules have moved from the gaps between nanopillars to the top of the nanopillar during the simulation, this process is formulated as dewetting-like behavior based on Wenzel droplets. Meanwhile, Wang et al. confirmed that the potential energy barrier to be overcome for the dewetting transition will increase with the increased the interpillar spacing (the contact area of solid-liquid interface increases). Based on this, for the surfaces P_0.25_S_0.25_D_3_ and P_0.4_S_0.25_D_3_, there was no tendency of droplet transition from the Wenzel state to the Cassie-Baxter state as the condensed droplet volume increased (no dewetting-like behavior was observed).

For the surface P_0.25_S_0.4_D_2_, at the beginning of condensation, water molecules condensed into a water film of about two–three water molecules thickness in the gaps between the structure. Subsequently, the water film thickness increases and becomes larger than the nanopillar height. The condensed water above the nanopillar changes into a spherical crown shape to achieve a stable state. In this stage, due to the weak restriction of water molecules by hydrophobic nanopillars, the water molecules in the upper part of the gaps are easily attracted by the crown condensate and leave the gap; on the contrary, the water molecules in the lower part of the gaps remain in the gap due to the strong attraction of the hydrophilic substrate to these water molecules. Since some water molecules move from the gaps to the top of the nanopillar during the simulation, this process is formulated as dewetting-like behavior based on liquid film. However, for the surfaces P_0.4_S_0.4_D_2_ and P_0.4_S_0.25_D_2_, the hydrophilic nanopillars strongly attract the water molecules in the upper part of the gaps, so that they remain in the gap, and therefore no dewetting-like behavior occurs. Moreover, for the surface P_0.25_S_0.4_D_3_, almost all the water molecules condensed in the enlarged gaps without staying at the top of the nanopillar, so no dewetting-like behavior occurred either.

In general, when film condensation occurs, the thermal resistance is mainly determined by the liquid film thickness [48]. The dewetting-like behavior based on liquid film observed in this work makes the water film filled in the gap thinner, which reduces the thermal resistance to some extent at the initial stage of the condensation. In addition, when dropwise condensation occurs, the thermal resistance decreases significantly after the condensed droplets shed off the surface [48]. The dewetting-like behavior based on Wenzel droplets observed in this work helps the condensed droplets to reduce the water molecules in the gaps, resulting in a smaller contact area between the droplets and the surface. This means that the interaction between the droplets and the surface weakens, which is beneficial for the condensate droplets to shed off the surface. Therefore, it is considered that the dewetting-like behavior based on Wenzel droplets has a potential role in reducing thermal resistance at the initial stage of the condensation.

### 3.4. Condensation on the Hydrophobic Nanopillar Surfaces with Hydrophilic Tops

**Figure 12:**
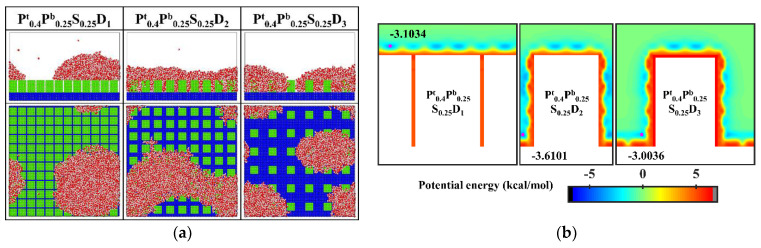
(**a**) The snapshot of the condensate formed on the surfaces P^t^_0.4_P^b^_0.25_S_0.25_D_i_ at the end of the simulation (**b**) Equipotential curves of potential energy between water molecule and the nanopillar surfaces (P^t^_0.4_P^b^_0.25_S_0.25_D_i_). The minimum values of the potential energy are given in the figure and their positions are marked with a pink dot.

In order to improve the condensation efficiency, controlling the vapor condensation process into the dropwise condensation mode that generates Cassie-Baxter droplets has received focused attention. As mentioned in Section 3.1.2, reducing the interpillar spacing or the surface energy is beneficial to controlling the condensation mode of water vapor on the surface into the Cassie-Baxter DWC mode. Additionally, inspired by the Namib desert beetle, some nanostructured surfaces with hybrid wettability have recently been applied to control the condensation of water vapor into Cassie-Baxter droplets on the surface [25,36]. Hou et al. designed a hybrid surface with patterned high-contrast wettability (micropillars with a diameter of 6 μm), which allows water vapor to nucleate regularly and condense into Cassie-Baxter droplets [25]. Gao et al. proposed a nanopillar surface with hybrid wettability (nanopillar with width of 2.35 nm), which combined the advantages of the hydrophilic surfaces and hydrophobic surfaces to generate desired Cassie-Baxter droplets [36]. This excellent performance is attributed to the proper distribution of hydrophilic and hydrophobic regions on the surface. Unfortunately, as discussed in Section 3.1.3, the Cassie-Baxter DWC mode is not observed on the nanopillar surfaces with hybrid wettability, except when the interpillar spacing is D_1_. Here, in order to condense water vapor into Cassie-Baxter droplets that can easily shed off the surface, the wettability distribution of the nanopillar surface is redesigned, inspired by the biological water collection surface. Specifically, the vapor condensation on the hydrophobic nanopillar surfaces with hydrophilic tops is investigated. Based on the model in the present work, the surface wettability distribution is redefined such that only the upper part of the nanopillar of about 1/4 H is defined as hydrophilic, while the rest of the nanopillar and the substrate are both hydrophobic. Correspondingly, these surfaces are denoted as P^t^_0.4_P^b^_0.25_S_0.25_D_i_. The final morphology of the condensate on these surfaces is shown in Figure 12a. When the interpillar spacing is D_1_, the Cassie-Baxter state condensed droplet is observed on the surface. When the interpillar spacing is D_2_ and D_3_, the water vapor condensed on the surface as droplets in the Wenzel state. Figure 12b shows the equipotential curves of potential energy in the cases of P^t^_0.4_P^b^_0.25_S_0.25_D_i_. The droplet is in the Cassie-Baxter state when the interpillar spacing is D_1_, mainly due to the spatial restriction of water molecules by the gaps (the energy barrier makes it impossible for water molecules to enter the gaps). When the interpillar spacing is D_2_, the low potential energy region in the gap induces water molecules to stay, resulting in the formation of Wenzel droplets. When the interpillar spacing is further increased to D_3_, even though the potential energy in the gap is not as low as at D_2_, the area where the substrate is in contact with water molecules becomes larger, which raises the probability of condensation of water molecules in the gaps and thus induces the formation of Wenzel droplets. Obviously, these results are disappointing because previous studies have shown that the nanostructured surfaces with similar wettability distribution can promote the Cassie-Baxter mode nucleus [36,37]. Such as, Cassie-Baxter droplet is observed on the nanopillar surfaces with hybrid wettability (nanopillar with width of 2.35 nm) by Gao et al. [36]; Ding et al. designed a series of nanopillar surfaces with different hybrid wettability ratios (nanopillar with radius of 1 nm), and water vapor condensed into Cassie-Baxter droplets on many surfaces [37].

**Figure 13:**
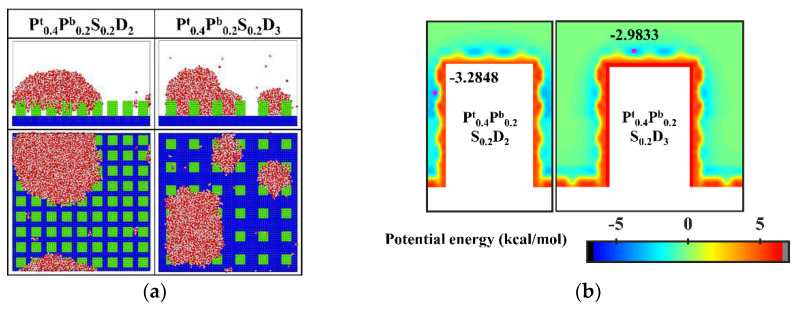
(**a**) The snapshot of the condensate formed on the surfaces at the end of the simulation in the cases of P^t^_0.4_P^b^_0.2_S_0.2_D_2_ and P^t^_0.4_P^b^_0.2_S_0.2_D_3_. (**b**) Equipotential curves of potential energy between water molecule and the nanopillar surfaces (P^t^_0.4_P^b^_0.2_S_0.2_D_2_ and P^t^_0.4_P^b^_0.2_S_0.2_D_3_). The minimum values of the potential energy are given in the figure and their positions are marked with a pink dot.

Since the condensation process on the aforementioned nanopillar surfaces with hydrophilic tops is different from the design prediction, the surface wettability distribution is further optimized. Specifically, 0.2 kcal mol^−1^, an interaction parameter indicating stronger hydrophobicity, is adopted to define the hydrophobic part of the nanopillar surface. Accordingly, two supplementary nanopillar surfaces with hydrophilic tops are modeled and noted as P^t^_0.4_P^b^_0.2_S_0.2_D_2_ and P^t^_0.4_P^b^_0.2_S_0.2_D_3_, respectively. The final morphology of the condensate and the equipotential curves of potential energy in these supplementary simulations are shown in Figure 13. Obviously, the condensed droplet is in the Cassie-Baxter state on the surface P^t^_0.4_P^b^_0.2_S_0.2_D_2_, while the water vapor still condenses into droplets in the Wenzel state on the surface P^t^_0.4_P^b^_0.2_S_0.2_D_3_. As shown in Figure 13b, the enhanced hydrophobicity of the hydrophobic part of the surface leads to a rise in potential energy at the bottom of the gap, causing water molecules to be preferentially deposited near the top of the nanopillar. When the interpillar spacing is D_2_, the higher potential energy at the bottom of the gap prevents the condensed droplets from sinking into the gap, so the droplets are in the Cassie-Baxter state. However, when the interpillar spacing increases to D_3_, the widened gap allows more water molecules to condense around the nanopillar, which makes it easier for the condensed droplets to penetrate into the structure, resulting in the formation of Wenzel droplets.

In addition, almost all water molecules condensed on the bionic surface P^t^_0.4_P^b^_0.2_S_0.2_D_2_ during the simulation. Compared to Figure 6a, although the condensation process is all in the Cassie-Baxter DWC mode, the condensation rate on this bionic surface is significantly faster than that on the hydrophobic surface with uniform wettability. Overall, these results verify that this biomimetic design of the surface wettability distribution is an effective way to control the condensation mode of water vapor on the surface into the dropwise condensation mode that generates Cassie-Baxter droplets. However, suitable parameters need to be chosen for the design. If the hydrophobicity of the hydrophobic part of the surface is weak or the interpillar spacing of the surface is too large, the condensation process can hardly be the Cassie-Baxter DWC mode.

## 4. Conclusions

In summary, the initial stage of the water vapor condensation process on various nanopillar surfaces is visualized by MD simulation, and the coupling effect of the geometric parameters and wettability distribution of the surface on the condensation has been investigated systematically at the nanoscale. The key findings obtained in this work can be summarized as follows:

- Three typical condensation modes are observed on nanopillar surfaces, namely Cassie-Baxter DWC mode, Wenzel DWC mode, and FWC mode, respectively.
- The condensation mode is primarily determined by the nanopillar wettability when the nanopillars are densely distributed, while the substrate wettability mainly affects the condensation mode when the nanopillars are sparsely distributed.
- The effective contact area fraction is proposed, which more accurately reflects the influence of geometric parameters on the condensation rate of the nanopillar surface at the nanoscale compared to the surface solid fraction. Specifically, the condensation rate of the nanopillar surface increases with the increase of the effective contact area fraction.
- The effect of wettability distribution on condensation rate differs for nanopillar surfaces with different geometric parameters.
- For efficient condensation, three surface design methods are summarized, which can control the condensation mode of water vapor on the surface into the dropwise condensation mode that generates Cassie-Baxter droplets. Specifically, the design guidelines are smaller nanostructure spacing, lower surface energy, and suitable surface wettability distribution (such as hydrophobic nanopillar surfaces with hydrophilic tops), respectively.

These findings contribute to the understanding of the effect of surface parameters on the condensation, and guide the design of nanopillar surfaces used to control condensation behavior.

## Figures and Tables

**Table 1:** Subscript notation for nanopillar wettability, substrate wettability, and interpillar spacing.

Subscript	Nanopillar Wettability (P)	Substrate Wettability (S)	Interpillar Spacing (D)
0.25	98°	98°	/
0.4	38°	38°	/
1	/	/	3.615 Å
2	/	/	7.23 Å
3	/	/	14.46 Å

**Table 2:** The surface solid fraction, the effective contact area fraction and the surface roughness of nanopillar surfaces with different interpillar spacing.

Interpillar Spacing (D)	Surface Solid Fraction (*ϕ*)	Effective Contact Area Fraction (*η*)	Surface Roughness * (*r*)
3.615 Å	0.444	0.098	4.556
7.23 Å	0.250	0.750	3.000
14.46 Å	0.111	0.529	1.889

* The surface roughness, $r\;=\;\left(a^{2}\;+\;D_{i}^{2}\;+\;2aD_{i}\;+\;4aH\right)/{\left(a\;+\;D_{i}\right)}^{2}$.

## Data Availability

The original contributions presented in the study are included in the article, and further inquiries can be directed to the corresponding author.

## Supplementary Materials

The following supporting information can be downloaded at: https://www.mdpi.com/article/10.3390/mi14010050/s1, Supplementary materials. Figure S1: View of initialized water box on a flat surface. Figure S2: The snapshot of the droplet on different surface. Figure S3: Time-lapse images of the condensation process on nanopillar surface with the interpillar spacing of D1. Figure S4: Time-lapse images of the condensation process on nanopillar surface with the interpillar spacing of D_2_. Figure S5: Time-lapse images of the condensation process on nanopillar surface with the interpillar spacing of D_3_. Figure S6: Time-lapse images of the condensation process on nanopillar surface with energy parameter of 0.2 kcal mol^−1^. Figure S7: Time-lapse images of the condensation process on the hydrophobic nanopillar surfaces with hy-drophilic tops. Ref. [50] are mentioned in the supplementary materials.
