# Supplementary material for: Design of Nanostructured Surfaces for Efficient Condensation by Controlling Condensation Modes
Source: Micromachines (Basel). 2022 Dec 25;14(1):50. doi: 10.3390/mi14010050 (PMC9864459; doi:10.3390/mi14010050)
Supplement: Supplementary file 1 [file micromachines-14-00050-s001.zip › micromachines-2081107-supplementary.pdf]

The geometric parameters of each component in the simulation system

The water density of the system corresponds to the saturated water vapor density at 500 K.

The thicknesses of the hot flat surface and the substrate of nanopillar surface are 10.845 Å and 7.23 Å, respectively.

The LJ potential parameters and atomic charges used in the simulation system

The LJ parameters of the nanopillar surface and the hot flat surface are $\varepsilon_{Cu\text{-}Cu}$ = 4.72 kcal mol^{-1} and $\sigma_{Cu\text{-}Cu}$ = 2.616 Å. It should be noted that there is no interaction potential between the nanopillar surface and the hot flat surface, so these two surfaces do not affect each other under the periodic conditions in the *z* direction.

In the TIP4P-Ew water model, an additional M site is used to carry the partial charge of oxygen atoms, and this site M is located at a fixed distance (r_{OM}) away from the oxygen along the bisector of the H-O-H bond angle. The full TIP4P-Ew force field parameters are $\varepsilon_{O\text{-}O}$ = 0.16275 kcal mol^{-1}, $\sigma_{O\text{-}O}$ = 3.16435 Å, r_{OM} = 0.125 Å, q_O = 0 e, q_H = 0.5242 e, and q_M = -1.0484 e.

The method used to obtain the contact angles of water droplet

To confirm the wettability of the surface, the contact angles of water droplet on different flat surfaces are obtained by MD simulations. The initial water box (consist of 8000 water molecules) is placed on the surface as shown in Figure S1, and it will evolve into droplets with different contact angles due to the different wettabilities of surface. The simulation is carried out for 1 ns at 300 K.

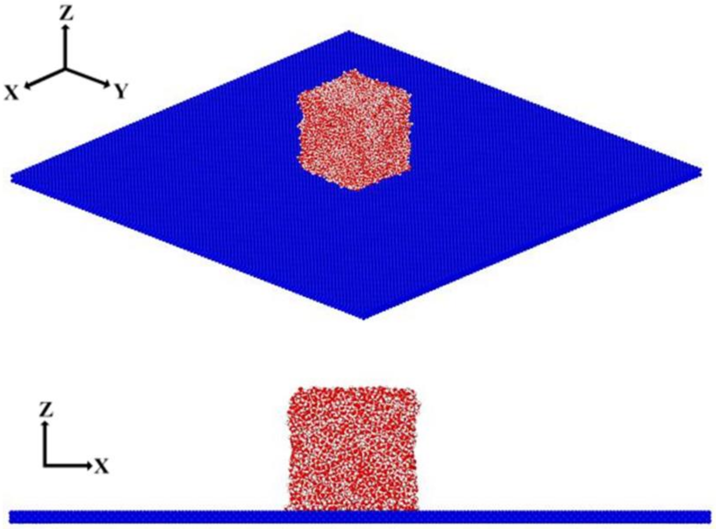

Figure S1. View of initialized water box on a flat surface.

A popular circular method is applied to determine the contact angle [50]. First, the droplet is divided into many layers in the vertical direction, and the positions of outermost water molecules of every single horizontal layer are found. Second, since the droplets can ignore the influence of gravity at the nanoscale, a circular best fit through these points is obtained by the least square method and the contact angle is measured. The contact angles of water droplet on different graphene are shown in Figure S2. Obviously, the contact angle decreases as the energy parameter $\varepsilon_{water\text{-}Cu}$ increases.

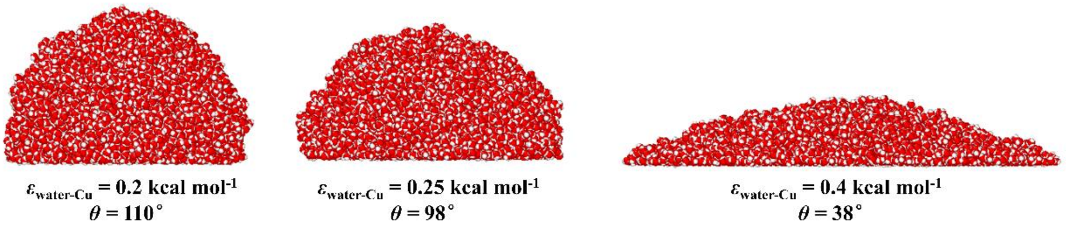

Figure S2. The snapshot of the droplet on different surface.

Snapshots of condensation process on nanopillar surfaces

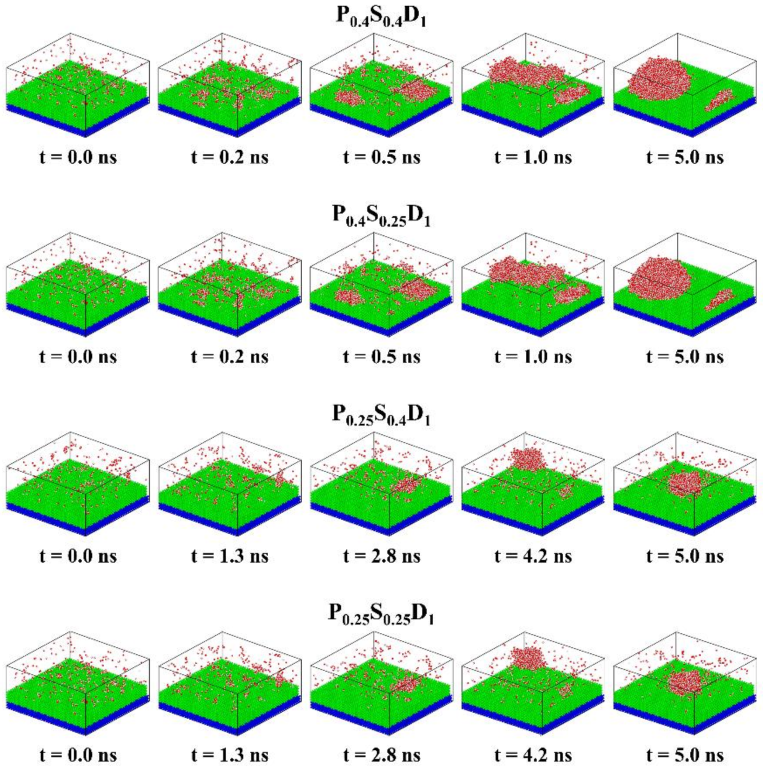

Figure S3. Time-lapse images of the condensation process on nanopillar surface with the interpillar spacing of D_1.

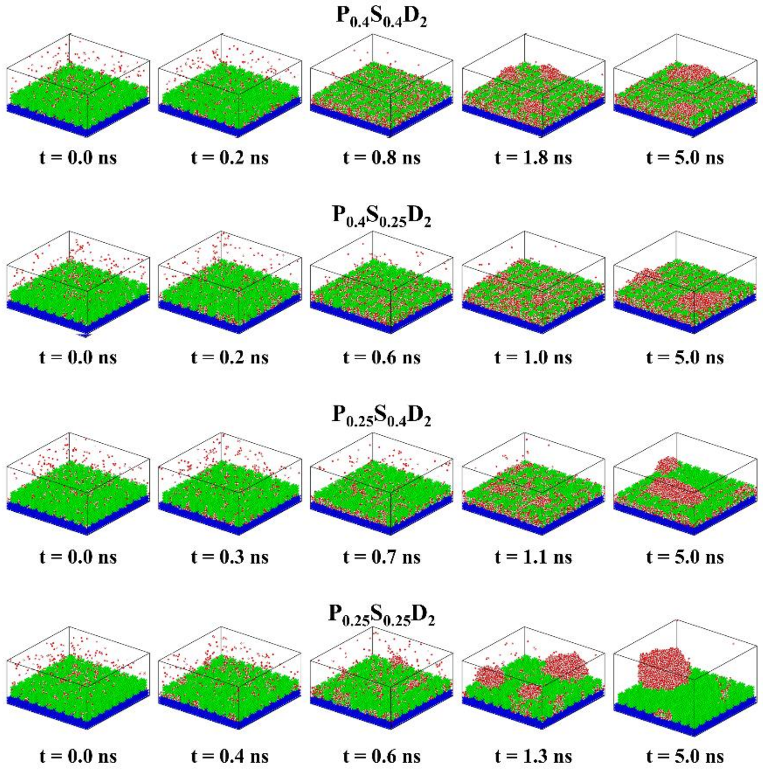

Figure S4. Time-lapse images of the condensation process on nanopillar surface with the interpillar spacing of D_2.

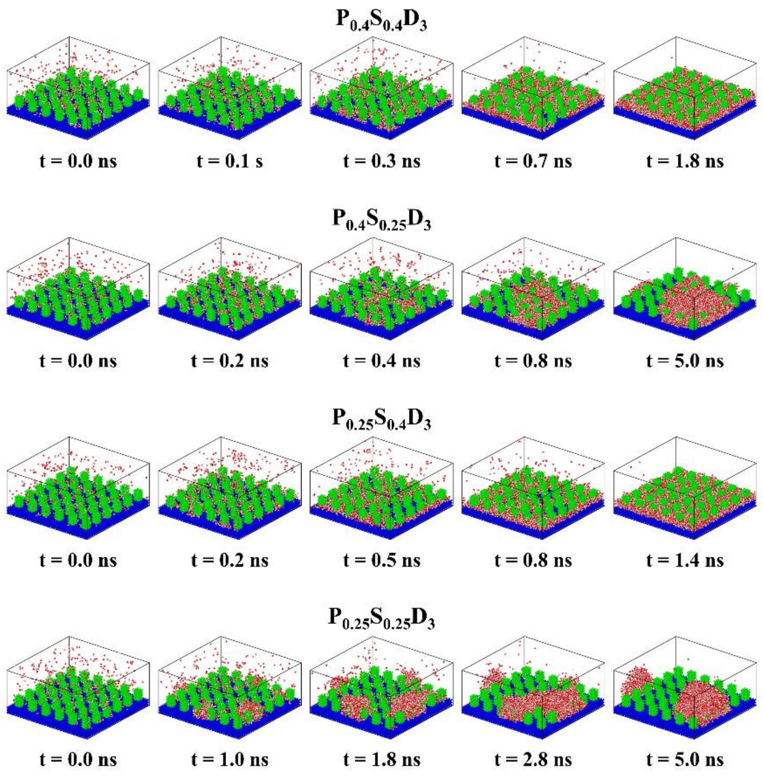

Figure S5. Time-lapse images of the condensation process on nanopillar surface with the interpillar spacing of D_3.

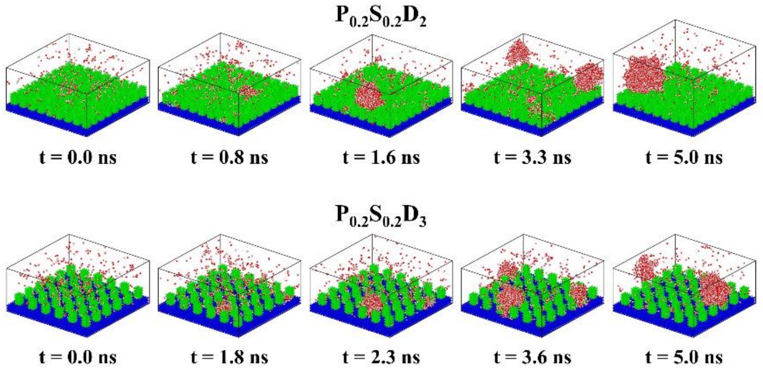

Figure S6. Time-lapse images of the condensation process on nanopillar surface with energy parameter of 0.2 kcal mol^{-1}.

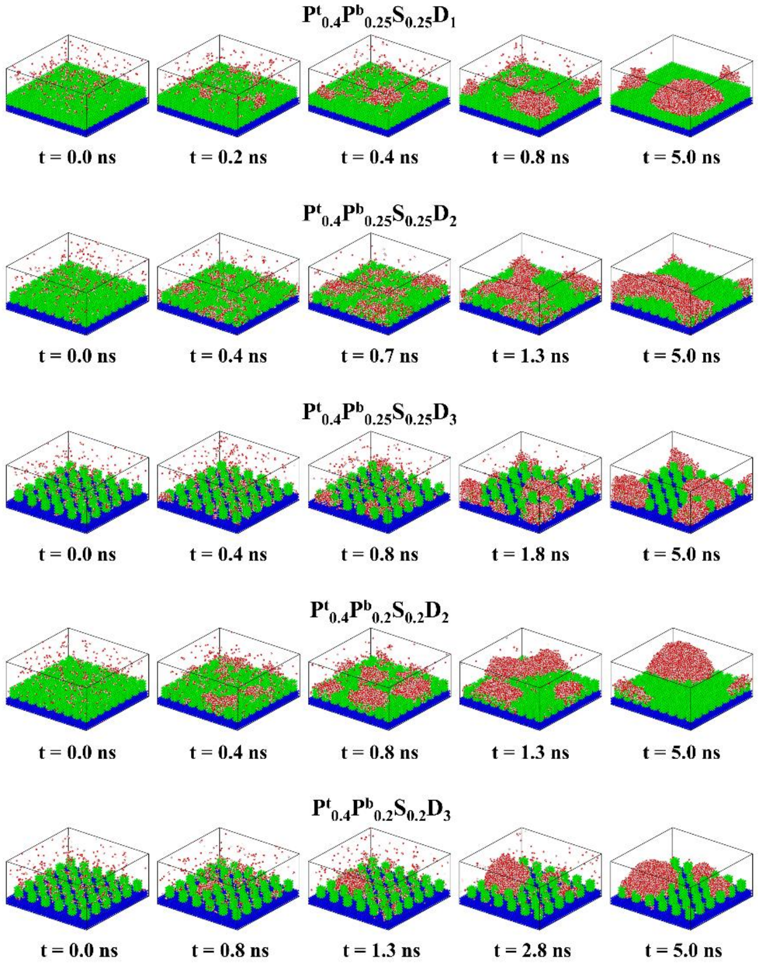

Figure S7. Time-lapse images of the condensation process on the hydrophobic nanopillar surfaces with hydrophilic tops.

References

50. K. Zhang, F. Wang, X. Zhao, The self-propelled movement of the water nanodroplet in different surface wettability gradients: A contact angle view, Computational Materials Science, 124 (2016) 190-194.
